# The association between hypertensive disorders during pregnancy and maternal and neonatal outcomes: a retrospective claims analysis

**DOI:** 10.1186/s12884-023-05818-9

**Published:** 2023-07-14

**Authors:** Samantha G. Bromfield, Qinli Ma, Andrea DeVries, Tiffany Inglis, Aliza S. Gordon

**Affiliations:** 1 Health Services Research, Elevance Health, Indianapolis, IN USA; 2 Enterprise Clinical Operations, Elevance Health, Indianapolis, IN USA

**Keywords:** Hypertension, Pregnancy, Outcomes, Neonatal, Maternal, Preeclampsia

## Abstract

**Background:**

Hypertensive disorders during pregnancy continue to increase in prevalence and are associated with several adverse outcomes and future cardiovascular risk for mothers. This study evaluated the association of hypertensive disorders compared to no hypertension during pregnancy with neonatal and maternal outcomes. We then evaluated risk factors associated with progression from a less to more severe hypertensive disorder during pregnancy.

**Methods:**

We conducted a propensity-matched retrospective cohort study utilizing Medicaid claims data from a national insurer. The study population consisted of mothers with and without hypertensive disorders who delivered between 7/1/2016–12/31/2018 and their infants. Hypertensive disorders included gestational hypertension, chronic hypertension, preeclampsia, and superimposed preeclampsia. Propensity score matching was used to match mothers without to those with hypertensive disorders. Regression models were used to compare maternal and neonatal outcomes. Stepwise logistic regression was used to determine characteristics associated with the progression of gestational hypertension to preeclampsia or chronic hypertension to superimposed preeclampsia.

**Results:**

We observed the highest risk of cesarean delivery (odds ratio [OR]:1.61 and 1.99) in mothers and preterm delivery (OR:2.22 and 5.37), respiratory distress syndrome (OR:2.39 and 4.19), and low birthweight (OR:3.64 and 9.61) in babies born to mothers with preeclampsia or superimposed preeclampsia compared to no hypertension, respectively (*p* < 0.05 for all outcomes). These outcomes were slightly higher among chronic or gestational hypertension compared to no hypertension, however, most were not statistically significant. Risk of neonatal intensive care unit utilization was higher among more severe hypertensive disorders (OR:2.41 for preeclampsia, OR:4.87 for superimposed preeclampsia). Obesity/overweight and having a history of preeclampsia during a prior pregnancy were most likely to predict progression from gestational/chronic hypertension to preeclampsia/superimposed preeclampsia.

**Conclusion:**

Mothers and neonates born to mothers with preeclampsia or superimposed preeclampsia experienced more adverse outcomes compared to those without hypertension. Mothers and neonates born to mothers with gestational hypertension had outcomes similar to those without hypertension. Outcomes for those with chronic hypertension fell in between gestational hypertension and preeclampsia. Obesity/overweight and having a history of preeclampsia during a prior pregnancy were strong risk factors for hypertension progression.

**Supplementary Information:**

The online version contains supplementary material available at 10.1186/s12884-023-05818-9.

## Background

Hypertensive disorders during pregnancy are relatively common, affecting 1 in every 10 pregnancies in the United States, and have been increasing in prevalence since the 1970’s [1–3]. Hypertensive disorders during pregnancy include several conditions that are defined according to the history of maternal blood pressure (BP), clinical features, and when during pregnancy it occurs. Hypertension beginning before 20 weeks gestation is considered chronic hypertension, after 20 weeks is considered gestational hypertension, and preeclampsia is distinguished by signs of damage to another organ, mostly the liver and kidneys. If a woman with chronic hypertension develops preeclampsia after 20 weeks, it is referred to as superimposed preeclampsia [3, 4].

Hypertensive disorders during pregnancy have been shown to be associated with adverse perinatal outcomes for both mother and baby. Chronic hypertension, superimposed preeclampsia, and preeclampsia are all associated with increased risk for stillbirth, preterm delivery, low birth weight, neonatal intensive care unit (NICU) admission, and cesarean delivery [2, 3, 5–10]. Hypertension during pregnancy is also a risk factor for postpartum preeclampsia [11].

Hypertensive disorders during pregnancy may adversely affect the expectant mother’s cardiovascular health, even after the end of pregnancy [12–20]. A meta-analysis of 27 studies showed that women with a history of preeclampsia during pregnancy showed signs of altered cardiac structure and function [15]. Another study reported an odds ratio (OR) of 2.16 for future ischemic heart disease, 1.94 for stroke, and 2.76 for renal disease for women who experience preeclampsia or gestational hypertension during pregnancy compared to those who remained normotensive [16]. Additionally, hypertensive disorders during pregnancy increase the risk of hypertension during the years after the pregnancy; studies have reported substantial persistence of hypertension during the first year postpartum [21, 22], as well as significantly higher rates of hypertension during the ten years post-pregnancy [16, 21, 23].

There are few studies comparing outcomes for hypertensive disorders in mothers enrolled in Medicaid as most of these studies are population-based, which often do not report insurance coverage of participants. As of 2020, Medicaid covered 42% of births in the U.S. [24]. Greiner et al. compared hypertensive disorders during pregnancy by insurance type and found that mothers with Medicaid had more severe BP and neonates with more NICU admissions and preterm birth [25]. Providing a better perspective of how different hypertensive disorders impact maternal and neonatal outcomes using a large Medicaid population would provide better insight on prevention and treatment efforts for this population. Therefore, we sought to use data collected from Medicaid-insured expectant mothers to evaluate the association between specific hypertensive disorders during pregnancy – gestational hypertension, chronic hypertension, preeclampsia, and superimposed preeclampsia – versus no hypertension during pregnancy and maternal and neonatal outcomes. Outcomes of interest included stillbirth, cesarean delivery, postpartum preeclampsia, and post-delivery hypertension for the mothers; preterm delivery, neonatal morbidities, and NICU admissions and costs for the babies; and the mothers’ healthcare utilization and cost in the year after delivery. We also evaluated demographic and clinical risk factors associated with progression to a more severe hypertensive disorder, which included progression from chronic hypertension to superimposed preeclampsia or from gestational hypertension to preeclampsia.

## Methods

### Data source and ethics

We conducted a retrospective cohort study using Medicaid data from the Healthcare Integrated Research Database (HIRD). The HIRD contains medical and pharmacy claims from a large health insurer with health plans across the United States. This study was in full compliance with the Health Insurance Portability and Accountability Act. Review by the institutional review board was not required as it was not considered human subject research and only a limited dataset with no patient identifiers was used.

### Study participants

We utilized a cohort of mothers with Medicaid data from 14 states across the United States (US). We identified expectant mothers who delivered or whose pregnancy ended between July 1, 2016 and December 31, 2018. This date was defined as the index date. If a mother experienced more than one pregnancy during this time, only the first pregnancy was included. We included mothers who met the following criteria: had continuous medical and pharmacy enrollment in their health plan on or before week 16 of pregnancy until delivery or the end of the pregnancy, were 13 to 55 years of age at delivery, the pregnancy continued beyond 20 weeks of gestation, and had an inpatient delivery or hospitalization on the index date. Once the study sample was identified, we linked the records of babies born to their mother. The analyses for all neonatal outcomes were restricted to liveborn singleton babies who were linked to their mothers.

### Exposure

Hypertension during pregnancy was defined as having at least one medical claim with a diagnosis of a hypertensive disorder (see code list in Supplemental Table 1), from the estimated start of gestation to the end of pregnancy. Mothers with claims for gestational hypertension, chronic hypertension, preeclampsia, and superimposed preeclampsia were included and classified based on the most severe type of hypertension they experienced during their pregnancy. We used the following hierarchy to assign mothers into a hypertension category: superimposed preeclampsia, preeclampsia, chronic hypertension, gestational hypertension, and no hypertension. Mothers with eclampsia (< 1%) were removed from our sample. For our first objective, mothers who experienced a hypertensive disorder during pregnancy were compared to mothers who did not have hypertension during pregnancy. This resulted in four comparisons: gestational hypertension, chronic hypertension, preeclampsia, and superimposed preeclampsia each compared to no hypertension.

For our second objective, we evaluated the progression of a hypertensive disorder to a more severe type during pregnancy. Specifically, we looked at those who progressed from gestational hypertension to preeclampsia and those who progressed from chronic hypertension to superimposed preeclampsia. These mothers who progressed were compared to those who did not progress. Progression was defined as having at least one claim for gestational or chronic hypertension and at least one claim for the more severe hypertensive disorders, preeclampsia or superimposed preeclampsia, respectively. Mothers who did not progress were defined as having claims for gestational or chronic hypertension only.

### Outcomes

Primary outcomes were measured in claims for both mothers and neonates; code lists for all outcomes may be found in the Supplement. The neonate was the unit of analysis for neonatal outcomes and the expectant mother was the unit of analysis for the maternal outcomes. Neonatal outcomes included pre-term delivery, neonatal morbidities (including sepsis, respiratory distress syndrome, low birth weight, and severe congenital defects), and neonatal intensive care unit (NICU) admission, length of stay, and cost. Neonatal morbidities were measured within 28 days of delivery, whereas NICU variables were measured for the full NICU stay. Maternal outcomes included stillbirth and cesarean delivery, which were measured on the index date. Other maternal outcomes included postpartum preeclampsia – among the gestational and chronic hypertension cohorts, and post-delivery hypertension – among the gestational hypertension and preeclampsia cohorts. Postpartum preeclampsia was not measured among the cohorts with preeclampsia or superimposed preeclampsia during pregnancy in case claims for preeclampsia after delivery were related to the preeclampsia during pregnancy that had not yet resolved. Post-delivery hypertension was not measured among the cohorts with chronic hypertension or superimposed preeclampsia, as those cohorts had hypertension prior to pregnancy, so we would expect them to continue to have hypertension after delivery. Postpartum preeclampsia, post-delivery hypertension, and maternal healthcare utilization and cost in the year following delivery were measured among the subset of mothers who had 12 months of continuous health plan enrollment following the index date.

### Statistical analyses

#### Matching

A mother without a hypertensive disorder was matched via one-to-one propensity score matching (PSM) to each mother with a hypertensive disorder. PSM was conducted separately for each of the four hypertensive disorders. PSM was performed using multivariate logistic regression to estimate the association of having hypertensive disorder with baseline characteristics including age, race, region, socioeconomic status (at the census block group level), presence of prenatal care during the first trimester, Deyo-Charlson Index score, complications during pregnancy, history of hypertensive disorders during a pregnancy prior to the index pregnancy, and other comorbid conditions (see Supplemental Table 2). All covariates were identified in claims and health plan enrollment data; code lists for all covariates may be found in the Supplement. Each matched hypertensive disorder group was selected using a greedy algorithm to ensure baseline comparability of the two groups after matching.

#### Outcomes comparison

Logistic regression models were used to calculate odds ratios (ORs) for neonatal and maternal outcomes associated with each hypertensive disorder during pregnancy compared to no hypertension. Poisson regression models were used to calculate the incidence rate ratios (IRR) for healthcare utilization variables. Gamma regression models were used to calculate the mean difference in cost variables associated with having compared to not having a hypertensive disorder. Having no hypertension was used as the reference categories in all regression analyses. Since matched groups were balanced on all covariates (standardized mean difference < 0.10), no covariates were included in the regressions. *P*-values < 0.05 were considered statistically significant.

#### Predicting progression

We used multivariate logistic regression with forward stepwise selection to determine demographic and clinical characteristics associated with the progression of gestational or chronic hypertension to preeclampsia or superimposed preeclampsia during the pregnancy. Forward stepwise selection with a cutoff of *p* = 0.30 for inclusion was used to reduce the number of covariates in the final model. The order of appearance in the stepwise model was used as an indicator to rank the strength of the association between the risk factors and progression, since the model first identifies the variable that accounts for the most variation. The same baseline characteristics that were used for PSM in the first objective were included as predictors in these models (see the full list of baseline characteristics in Supplemental Table 2).

All statistical analyses were performed using SAS Enterprise Guide 7.1 (SAS Institute Inc., Cary, NC).

## Results

### Study cohort

We identified 140,425 expectant mothers who met our study inclusion criteria. The mean (SD) age was 27 (5.8) years and 28.4% (*n* = 39,839) were black. Of these mothers, 113,934 (81.1%) did not have hypertension, 11,845 (8.4%) had gestational hypertension, 3,460 (2.5%) had chronic hypertension, 9,305 (6.6%) had preeclampsia, and 1,881 (1.3%) had superimposed preeclampsia. Our one-to-one PSM algorithm yielded four sets of matched pairs: 11,583 for those with gestational versus no hypertension, 3,422 for those with chronic versus no hypertension, 9,234 for those with preeclampsia versus no hypertension, and 1,755 for those with superimposed preeclampsia versus no hypertension. Supplemental Table 2 shows the baseline demographic and clinical characteristics for these matched pairs. Additionally, Table 1 provides sample sizes for each subgroup that was analyzed, i.e., the sample of infants for neonatal outcomes and the sample of mothers with 12 months continuous health plan enrollment post-delivery for maternal post-delivery hypertension/preeclampsia, healthcare utilization, and healthcare cost.

**Table 1:** Sample sizes for each matched pair of hypertensive disorders during pregnancy and subgroups within each matched pair among expectant mothers in Medicaid

	No Hypertension	Gestational Hypertension	No Hypertension	Chronic Hypertension	No Hypertension	Preeclampsia	No Hypertension	Superimposed Preeclampsia
**Sample size among all (matched) mothers**	11,583	11,583	3422	3422	9234	9234	1755	1755
**Sample size among mothers with 12 months continuous eligibility**	4467	4528	1494	1553	3582	3678	713	751
**Sample size of liveborn and singleton neonates linked to mothers**	9511	9481	2816	2782	7515	7407	1440	1411

### Maternal outcomes

In the propensity-matched cohort, the risk of cesarean delivery was higher the more severe the hypertensive disorder, with preeclampsia and superimposed preeclampsia having the highest risk (OR:1.61, 95% CI:1.52–1.71 and OR:1.99, 95% CI:1.74–2.28, respectively) when compared to not having hypertension. For less severe forms of hypertension, there was a slightly elevated risk of cesarean delivery with an OR of 1.16 (95% CI:1.10–1.23) for gestational hypertension, and the risk for chronic hypertension compared with no hypertension was not significant with an OR of 1.08 (95% CI:0.98–1.19) (Table 2). The risk of stillbirth was highest in mothers with chronic hypertension (OR:1.64, 95% CI:1.12–2.41) and preeclampsia (OR:1.53, 95% CI:1.15–2.02) compared to those without hypertension, though this outcome was 2% or less among all groups. Among mothers with 12 months continuous enrollment after delivery, those with gestational (OR:3.52; 95% CI:2.21–5.60) or chronic hypertension (OR:3.76; 95% CI:1.63–8.66) had a substantially higher risk of postpartum preeclampsia, and those with gestational hypertension (OR:8.05; 95% CI:6.61–9.79) and preeclampsia (OR:8.01; 95% CI:6.39–10.03) had substantially higher risks of hypertension during the year following delivery.

**Table 2:** The association between hypertensive disorders during pregnancy and maternal and neonatal clinical outcomes among Medicaid mothers

	**Gestational Hypertension**		**Chronic Hypertension**		**Preeclampsia**		**Superimposed Preeclampsia**	
	**OR** **(95% CI)**	***P*-value**	**OR** **(95% CI)**	***P*-value**	**OR** **(95% CI)**	***P*-value**	**OR** **(95% CI)**	***P*-value**
**All matched mothers**^**a**^								
Cesarean delivery	1.16 (1.10-1.23)	<0.01	1.08 (0.98-1.19)	0.12	1.61 (1.52-1.71)	<0.01	1.99 (1.74-2.28)	<0.01
Stillbirth	1.27 (0.96-1.68)	0.10	1.64 (1.12-2.41)	0.01	1.53 (1.15-2.02)	<0.01	1.24 (0.70-2.22)	0.46
**Mothers with 12 months continuous enrollment, measured post-delivery**^**a**^								
Postpartum preeclampsia	3.52 (2.21-5.60)	<0.01	3.76 (1.63-8.66)	<0.01	-	-	-	-
Post-delivery hypertension	8.05 (6.61-9.79)	<0.01	-	-	8.01 (6.39-10.03)	<0.01	-	-
**Liveborn and singleton neonates linked to mothers, measured within 28 days of birth**^**a**^								
Pre-term delivery	0.97 (0.88-1.08)	0.61	1.19 (1.01-1.41)	0.04	2.22 (2.00-2.45)	<0.01	5.37 (4.29-6.73)	<0.01
Sepsis	1.05 (0.89-1.24)	0.56	1.73 (1.29-2.32)	<0.01	1.51 (1.26- 1.80)	<0.01	3.67 (2.41-5.59)	<0.01
Respiratory distress syndrome	1.11 (1.01-1.22)	0.03	1.28 (1.08-1.51)	<0.01	2.39 (2.16-2.64)	<0.01	4.19 (3.38-5.18)	<0.01
Low birth weight	1.00 (0.90-1.11)	0.99	1.39 (1.17-1.64)	<0.01	3.64 (3.29-4.02)	<0.01	9.61 (7.72-11.96)	<0.01
Severe congenital defects^b^	1.14 (0.88-1.47)	0.32	0.90 (0.58-1.38)	0.62	1.05 (0.79-1.38)	0.75	1.07 (0.60-1.90)	0.83

*OR* Odds ratio, were calculated from logistic regression models, *CI* Confidence Intervals. No Hypertension was the reference group for all comparisons

^a^ Sample size for each subgroup available in Table 1

^b^ Severe congenital defects include anophthalmia/microphthalmia, anotia/microtia, craniosynostosis, diaphragmatic hernia, Down Syndrome, esophageal atresia, gastroschisis, hypospadias, microcephaly and upper and lower limb reduction defects

### Neonatal outcomes

Among neonates linked to mothers in each matched pair, we observed that the risk of preterm birth was highest in those born to mothers with preeclampsia (OR:2.22, 95% CI:2.00-2.45) and superimposed preeclampsia (OR:5.37, 95% CI:4.29–6.73) compared to no hypertension (Table 2). This risk was not significant for those with gestational hypertension (OR:0.97, 95% CI:0.88–1.08) and slightly higher among mothers with chronic hypertension (OR:1.19, 95% CI:1.01–1.41). Of the neonatal morbidities, the risk for sepsis, respiratory distress syndrome, and low birth weight got progressively higher the more severe the hypertensive disorder compared to no hypertension, with the highest risks among those born to mothers with superimposed preeclampsia (OR:3.67, 95% CI:2.41–5.59; OR:4.19, 95% CI:3.38–5.18; and OR:9.61, 95% CI:7.72–11.96, for each outcome respectively). All types of hypertensive disorders had a significantly higher risk of these conditions, except for gestational hypertension, which only had a higher risk of respiratory distress syndrome (not sepsis or low birth weight).

### Maternal and neonatal utilization and cost

Among the subset of mothers with 12 months of continuous enrollment following the index date, having chronic hypertension or preeclampsia (*p* < 0.01) and possibly superimposed preeclampsia (*p* = 0.06) was associated with a higher odds of an emergency department (ED) visit, and preeclampsia (*p* < 0.01) and possibly chronic hypertension (*p* = 0.06) were associated with a higher odds of hospitalization (Table 3) after the index discharge. When compared to those without hypertension, the average pharmacy and medical cost were higher for each hypertensive disorder during pregnancy, except for chronic hypertension which had no difference in pharmacy cost compared with mothers without hypertension. The risk of NICU admission was highest for neonates born to mothers with preeclampsia (OR:2.41, 95% CI:2.21–2.63) and superimposed preeclampsia (OR:4.87, 95% CI:4.01–5.90) when compared to those born to mothers without hypertension. Neonates born to mothers with more severe hypertensive disorders (preeclampsia and superimposed preeclampsia) had higher mean NICU cost (mean difference:+$4,212, 95% CI: +$3,548 to +$4,940 and mean difference:+$13,163, 95% CI: +$9,992 to +$17,077, respectively) compared to those without hypertension. Note that this includes $0 cost for neonates who did not have a NICU stay. Among neonates with a NICU stay, those born to mothers with preeclampsia and superimposed preeclampsia had longer stays in the NICU (IRR:3.65, 95% CI:2.37–5.05 and IRR:8.87, 95% CI:5.37–13.04, respectively). For every maternal and neonatal healthcare utilization and cost outcome except pharmacy cost, the chronic hypertension cohort experienced greater increases in utilization and cost than the gestational hypertension cohort compared to the no hypertension cohort.

**Table 3:** The association between hypertensive disorders during pregnancy and maternal and neonatal healthcare utilization and cost among Medicaid mothers

	**Gestational Hypertension**		**Chronic Hypertension**			**Preeclampsia**		**Superimposed Preeclampsia**	
	**OR/IRR/** **Difference** **(95% CI)**	***P*-value**	**OR/IRR/** **Difference** **(95% CI)**		***P*-value**	**OR/IRR/** **Difference** **(95% CI)**	***P*-value**	**OR/IRR/** **Difference** **(95% CI)**	***P*-value**
**Maternal Post-Discharge Healthcare Utilization and Costs**									
**Healthcare Utilization**									
Hospitalizations (OR)	1.06 (0.93, 1.21)	0.40	1.23 (0.99, 1.53)		0.06	1.43 (1.24, 1.66)	<0.01	1.04 (0.75, 1.43)	0.83
Hospitalizations, count (IRR)	1.18 (1.05, 1.32)	0.01	1.29 (1.08, 1.53)		<0.01	1.58 (1.40, 1.78)	<0.01	1.19 (0.92, 1.53)	0.18
ED visits (OR)	1.06 (0.98, 1.15)	0.15	1.25 (1.08, 1.44)		<0.01	1.15 (1.05, 1.26)	<0.01	1.22 (1.00, 1.50)	0.06
ED visits, count (IRR)	1.11 (1.07, 1.16)	<0.01	1.32 (1.24, 1.40)		<0.01	1.09 (1.04, 1.13)	<0.01	1.03 (0.94, 1.13)	0.56
**Healthcare Cost**									
Total allowed pharmacy cost (mean difference)	$117 ($60, $179)	0.01	-$20 (-$172, $151)		0.81	$204 ($142, $271)	<0.01	$299 ($94, $541)	<0.01
Total allowed medical cost (mean difference)	$390 ($215, $575)	<0.01	$1,040 ($670, $1,447)		<0.01	$242 ($44, $454)	0.02	$581 ($79, $1,158)	0.02
**Neonatal Healthcare Utilization and Costs**									
NICU admission (OR)	1.21 (1.11-1.31)	<0.01	1.30 (1.12-1.51)	<0.01		2.41 (2.21-2.63)	<0.01	4.87 (4.01-5.90)	<0.01
NICU allowed cost (mean difference)	-$188 (-$422, -$67)	0.14	$4 (-$663, $784)	0.99		$4212 ($3548, $4940)	<0.01	$13,163 ($9,992, $17,077)	<0.01
NICU length of stay (mean difference), among those with a NICU admission	-2.26 (-3.16, -1.28)	<0.01	1.26 (-0.79, 3.63)	0.24		3.65 (2.37, 5.05)	<0.01	8.87 (5.37, 13.04)	<0.01

*OR* Odds ratio, were calculated from logistic regression models, *IRR* Incidence rate ratio, were calculated from Poisson regression, *CI* Confidence interval, *ED* Emergency department, *NICU* Neonatal intensive care unit. No Hypertension was the reference group for all comparisons

### Progression

From the population of 16,587 women with at least one claim for gestational hypertension, 4,742 (28.6%) had a claim on a later date for preeclampsia. Similarly, from the population of 5,081 women with at least one claim for chronic hypertension, 1,621 (31.9%) had a claim on a later date for superimposed preeclampsia.

The factors most likely to predict progression from gestational or chronic hypertension to preeclampsia or superimposed preeclampsia, respectively, are included in Table 4. We present the top 10 factors with *p* < 0.05 in the order of their entrance into the stepwise models. Obesity/overweight was one of the strongest predictors of progression to preeclampsia (second variable to enter the model, OR:1.29, 95% CI:1.20–1.39) or to superimposed preeclampsia (first variable to enter the model, OR:3.65, 95% CI:3.19–4.18). Having a history of preeclampsia (including preeclampsia, superimposed preeclampsia, or eclampsia) during a prior pregnancy was another top predictor of progression to preeclampsia (first variable to enter the model, OR:1.76, 95% CI:1.52–2.04) or superimposed preeclampsia (third variable to enter the model, OR:2.24, 95% CI:1.78–2.82). Having a history of gestational or chronic hypertension without progression to preeclampsia, compared to no evidence of hypertension during a prior pregnancy was associated with a lower risk of progressing from gestational hypertension to preeclampsia during the index pregnancy (OR:0.87, 95% CI:0.79–0.96), but put women with chronic hypertension at a higher risk of progression to superimposed preeclampsia (OR:1.59, 95% CI:1.37–1.85). Other significant risk factors for progression to more serious forms of hypertension included the presence of clinical conditions such as gestational diabetes (both conditions), urinary tract infection (preeclampsia, and a significant but not top 10 risk factor for superimposed preeclampsia), substance use disorder (superimposed preeclampsia), and history of postpartum hemorrhage (superimposed preeclampsia), among others. Demographics including age and race/ethnicity were also predictive of progression. Finally, having prenatal care start in the first trimester was associated with lower odds of developing superimposed preeclampsia (OR:0.70, 95% CI:0.61–0.80).

**Table 4:** Top 10 significant risk factors for progression to more severe hypertensive disorders during pregnancy among Medicaid mothers

**Step**	**Progression from Gestational Hypertension to Preeclampsia**			**Progression from Chronic Hypertension to Superimposed Preeclampsia**		
	**Risk Factor**	**OR (95% CI)**	***P*-value**	**Risk Factor**	**OR (95% CI)**	***P*-value**
**1**	Prior history of preeclampsia	1.76 (1.52-2.04)	<.0001	Obesity/overweight	3.65 (3.19-4.18)	<.0001
**2**	Obesity/overweight	1.29 (1.20-1.39)	<.0001	Gestational diabetes	3.63 (2.99-4.41)	<.0001
**3**	Urinary tract infection	1.19 (1.08-1.30)	<.0001	Prior history of preeclampsia	2.24 (1.78-2.82)	<.0001
**4**	Prior history of gestational hypertension without preeclampsia	0.87 (0.79-0.96)	0.003	Substance use disorder	1.88 (1.52-2.31)	<.0001
**5**	Gestational diabetes	1.18 (1.07-1.30)	0.003	History of postpartum hemorrhage	2.16 (1.55-3.00)	<.0001
**6**	Other race	1.12 (1.00-1.26)	0.025	Prenatal care in first trimester	0.70 (0.61-0.80)	<.0001
**7**	Age 26 to 35	0.93 (0.87-1.00)	0.030	Prior history of gestational or chronic hypertension without preeclampsia	1.59 (1.37-1.85)	<.0001
**8**				Diabetes	0.62 (0.49-0.78)	<.0001
**9**				Northeast region	0.54 (0.42-0.69)	<.0001
**10**				White race	0.71 (0.61-0.82)	<.0001

*OR* Odds ratio, *CI* Confidence interval

## Discussion

In this retrospective cohort study using claims data from 14 states across the US we found that mothers enrolled in Medicaid with preeclampsia or superimposed preeclampsia have worse outcomes for themselves and their babies than mothers without hypertension. Mothers with gestational hypertension had slightly higher risks of some poor outcomes compared to women without hypertension, but their outcomes were more similar to mothers without hypertension than to mothers with preeclampsia or superimposed preeclampsia. Those with chronic hypertension had higher risks for many poor outcomes such as preterm birth, sepsis, respiratory distress syndrome, and low birthweight compared to women without hypertension, but their risk was typically lower than mothers with preeclampsia or superimposed preeclampsia.

In contrast, while risks for stillbirth were highest among those with chronic hypertension, followed by preeclampsia, there was no statistical difference for those with gestational hypertension compared to those without hypertension. This may be due to mothers with chronic hypertension having a hypertensive disorder for a longer period, which leaves more time for a stillbirth to occur compared to many of the other hypertensive disorders in which delivery may occur sooner after diagnosis, particularly preeclampsia and superimposed preeclampsia, where induction is often indicated and delivery typically occurs very shortly after diagnosis. Hence, the daily risk of stillbirth following chronic hypertension diagnosis is unlikely to be higher than other more severe hypertensive disorders. Additionally, several prior studies have not only reported an association between chronic hypertension and stillbirth but have cited chronic hypertension as one of the most prevalent risk factors for stillbirth [5, 6, 26–29]. When compared to infants born to mothers with no hypertension, NICU admissions, preterm delivery, and low birth weight were most likely to occur in infants born to mothers with preeclampsia or superimposed preeclampsia, while those with chronic hypertension or gestational hypertension were only at a slightly elevated risk (and mothers with gestational hypertension did not have any added risk of preterm delivery).

The association between hypertensive disorders during pregnancy and adverse maternal or neonatal outcomes aligns with findings from previous research. A prior study of 1,417 pregnant women attending routine visits at two hospitals in the United Kingdom with a medical history of chronic hypertension found that chronic hypertension increased the risk of stillbirth, preeclampsia, small-for-gestational-age, gestational diabetes, preterm birth, and elective cesarean delivery compared to no hypertension [5]. Another study utilized a hospital perinatal database from the Washington University School of Medicine in Missouri to compare differences in outcomes for mothers with chronic hypertension (*n* = 1032), superimposed preeclampsia (*n* = 489) and preeclampsia (*n* = 4217) to those without any hypertensive disorder during pregnancy [8]. Tuuili et al. reported an increased risk for small-for-gestational age, NICU admission, preterm birth, cesarean delivery, and prolonged maternal hospital stay for mothers with hypertensive disorders during pregnancy compared to those without hypertension. Our study was unique in that we had a large sample of expectant mothers from across the US. Our study was also different in that we utilized Medicaid data, which services a demographic of women who are often socioeconomically disadvantaged and are at increased risk for many adverse conditions and outcomes [25].

In the current study, medical costs following birth were higher for mothers with any type of hypertension during pregnancy compared to no hypertension. We also found that the mean total cost for the NICU were the highest among infants born to mothers with preeclampsia or superimposed preeclampsia. While few studies have reported hypertension-related cost during pregnancy, Hao et al. compared costs among women with full term pregnancies with no complications, women with hypertension, and women with preeclampsia. Women with preeclampsia had the highest average incremental costs ($28,603). Most of the costs were related to the infants ($25,229 for infants compared to $3374 for mothers), which was driven by preterm delivery and other adverse events in the infants [30]. However, this study was limited to central and northeastern Pennsylvania, while our study utilized administrative data from a nationally representative sample of mothers with hypertensive disorders. Another study by Stevens et al. reported the national cost burden of preeclampsia to both mothers and infants. The cost of preeclampsia within the first 12 months after delivery were over $1 billion for mothers and $1 billion for infants in the US [31]. The cost burden of preeclampsia per infant ranged from $150,000 at 26 weeks gestational age to $1311 at 35 weeks gestational age [31]. Prevention of preeclampsia can help to reduce costs after delivery.

We took our study a step further by exploring factors that were associated with progressing to more severe hypertension during pregnancy (i.e., preeclampsia). This was important given the fact that BP typically decreases in the first and second trimester and then increases in the third, however for women with higher BP by the second trimester, changes in the cardiovascular system during pregnancy may contribute to the likelihood of more severe hypertension [32]. Two of the strongest risk factors for progression in our study were obesity/overweight and having a history of preeclampsia, superimposed preeclampsia, or eclampsia during a prior pregnancy, both of which are established risk factors for preeclampsia according to the ACOG [4]. In our study sample, more than 90% of those with a claim for obesity or overweight had a claim for obesity, as overweight may be less often coded in claims, particularly for pregnant women. Therefore, our findings related to this risk factor are mostly related to obesity rather than overweight. The obesity epidemic continues to rise among women of childbearing age in the US, with a prevalence that went from 25.1% to 2010 to 31.1% 2020 [33]. Increased BMI prior to pregnancy has been shown to be associated with higher mean systolic and diastolic BP at any point during pregnancy and postpartum [32]. Obesity is a strong risk factor for cardiovascular disease and has implications beyond pregnancy on cardiovascular health. UTI was also a strong predictor of progression to preeclampsia, though the connection between UTI and preeclampsia has remained unclear. However, one study suggested that perhaps the mechanisms involved includes aberrant placentation and systemic inflammation [34]. These risk factors for progression may be utilized by clinicians or health plans to target intervention and more aggressive treatment of chronic or gestational hypertension to attempt to prevent progression of disease to a more severe hypertension during pregnancy.

### Limitations

The findings from this study should be interpreted within the context of known and potential limitations. First, this was a retrospective, observational study which limits causal inference as it is subject to biases such as selection bias. While we matched patients on a number of demographic and clinical variables to create comparison groups with similar profiles, there still may have been unobserved differences between women with and without hypertension during pregnancy that may affect the outcomes we studied. Second, claims data lack detailed clinical measures such as laboratory values and vital signs such as BP, weight, and BMI which would help to further define the hypertensive disorders, covariates, and outcomes included in these analyses. Additionally, claims data may be subject to issues with accuracy or completeness, which might have introduced misclassification into our study. Third, we did not distinguish between preeclampsia with versus without severe features, and there are likely differences in outcomes between these groups. Fourth, we included several codes for “unspecified maternal hypertension” in our gestational hypertension cohort. While we assume that these codes are typically for gestational hypertension given the lack of prior codes for hypertension (chronic hypertension) or codes for more severe hypertensive disorders, this may lead to some misclassification bias in that cohort. Lastly, findings may not be generalizable to commercially insured members or individuals without insurance across the US.

## Conclusions

Hypertensive disorders during pregnancy continue to increase in prevalence, therefore this study addressed differences in outcomes between hypertensive disorders during pregnancy compared to no hypertension in mothers enrolled in Medicaid. We found that neonates and mothers had substantially more adverse outcomes when mothers had preeclampsia or superimposed preeclampsia. Babies born to mothers with chronic hypertension tended to have a higher risk of several adverse outcomes than mothers who developed gestational hypertension during pregnancy, however our findings show that this risk was still less than those mothers who developed preeclampsia or superimposed preeclampsia. Given pregnancy is a vulnerable period for both mother and baby, we also compared risk drivers of progressing to preeclampsia during pregnancy. Our findings showed that obesity and having a history of preeclampsia during a prior pregnancy were among the factors most likely to predict progression. These results can be used to inform clinicians and decision makers regarding prevention and treatment efforts for mothers at risk for or with hypertensive disorders during pregnancy. Lastly, these findings can be used by health plans to tailor outreach efforts for at-risk mothers, such as home blood pressure monitoring, to reduce some of the poor outcomes for these mothers and possibly prevent progression to preeclampsia.

## Supplementary Information

**Additional file 1: Supplemental Table 1.** ICD 10 Code list for hypertensive disorders during pregnancy. **Supplemental Table 2.** Baseline demographic and clinical characteristics for mothers with hypertensive disorders during pregnancy matched to those without hypertension in Medicaid mothers. **Supplemental Table 3.** Descriptive statistics for maternal and neonatal clinical outcomes, utilization and cost by maternal hypertensive disorder during pregnancy among Medicaid mothers (compared to mothers with no hypertension).

**Additional file 2.** Code lists Supplement.

## Data Availability

Requests for data should be directed to the corresponding author. The initial study was conducted for health care operations of a covered entity and the data was accessed under the HIPAA health care operations exception. The data used for this study included proprietary health claims and clinical data. Further information concerning data access would be provided upon request.
