# Supplementary material for: The association between hypertensive disorders during pregnancy and maternal and neonatal outcomes: a retrospective claims analysis
Source: BMC Pregnancy Childbirth. 2023 Jul 14;23:514. doi: 10.1186/s12884-023-05818-9 (PMC10347833; doi:10.1186/s12884-023-05818-9)
Supplement: Supplementary file 1 — Additional file 1: Supplemental Table 1. ICD 10 Code list for hypertensive disorders during pregnancy. Supplemental Table 2. Baseline demographic and clinical characteristics for mothers with hypertensive disorders during pregnancy matched to those without hypertension in Medicaid mothers. Supplemental Table 3. Descriptive statistics for maternal and neonatal clinical outcomes, utilization and cost by maternal hypertensive disorder during pregnancy among Medicaid mothers (compared to mothers with no hypertension). [file 12884_2023_5818_MOESM1_ESM.docx]

**Supplemental Table 1.** ICD 10 Code list for hypertensive disorders during pregnancy.

| **Hypertensive Disorder** | **ICD-10 Code List** |
| --- | --- |
| Chronic Hypertension | O10%, H35.031, H35.032, H35.033, H35.039, I10, I11.0, I11.9, I12.0, I12.9, I13.0, I13.10, I13.11, I13.2, I15.0, I15.1, I15.2, I15.8, I15.9, I67.4, |
| Gestational Hypertension | O13.2, O13.3, O13.4, O13.9, O16.1, O16.2, O16.3, O16.4, O16.9 |
| Preeclampsia | O14% |
| Superimposed Preeclampsia | O11.1, O11.2, O11.3, O11.4, O11.9 |

Note that all code lists used in the analyses are provided in the Code Lists Supplement.

**Supplemental Table 2.** Baseline demographic and clinical characteristics for mothers with hypertensive disorders during pregnancy matched to those without hypertension in Medicaid mothers.

| **Characteristics** | **No Hypertension** | | **Gestational hypertension** | | **No Hypertension** | | **Chronic hypertension** | | **No Hypertension** | | **Preeclampsia** | | **No Hypertension** | | **Superimposed Preeclampsia** | |
| --- | --- | --- | --- | --- | --- | --- | --- | --- | --- | --- | --- | --- | --- | --- | --- | --- |
|  | n / mean | % / SD | n / mean | % / SD | n / mean | % / SD | n / mean | % / SD | n / mean | % / SD | n / mean | % / SD | n / mean | % / SD | n / mean | % / SD |
| **Sample size, N** | 11583 |  | 11583 |  | 3422 |  | 3422 |  | 9234 |  | 9234 |  | 1755 |  | 1755 |  |
| **Age at delivery, in years (mean, SD)** | 26.69 | 5.75 | 26.96 | 6.08 | 28.9 | 5.96 | 28.81 | 6.03 | 26.24 | 5.83 | 26.49 | 6.21 | 29.91 | 5.98 | 29.83 | 5.99 |
| **Geographic region, n (%)** |  | | | | | | | | | | | | | | | |
| Northeast | 624 | 5% | 662 | 6% | 417 | 12% | 411 | 12% | 792 | 9% | 825 | 9% | 130 | 7% | 124 | 7% |
| Midwest | 1059 | 9% | 1,081 | 9% | 194 | 6% | 212 | 6% | 720 | 8% | 782 | 8% | 102 | 6% | 123 | 7% |
| South | 9104 | 79% | 8,998 | 78% | 2593 | 76% | 2584 | 76% | 7185 | 78% | 7,041 | 76% | 1410 | 80% | 1385 | 79% |
| West | 793 | 7% | 838 | 7% | 218 | 6% | 215 | 6% | 533 | 6% | 582 | 6% | 111 | 6% | 122 | 7% |
| **Individual Race (n,%)** |  | | | | | | | | | | | | | | | |
| White | 4780 | 41% | 4713 | 41% | 1183 | 35% | 1183 | 35% | 3321 | 36% | 3304 | 36% | 509 | 29% | 507 | 29% |
| Black | 4011 | 35% | 3935 | 34% | 1378 | 40% | 1351 | 39% | 3209 | 35% | 3122 | 34% | 822 | 47% | 798 | 45% |
| Hispanic | 1739 | 15% | 1815 | 16% | 449 | 13% | 460 | 13% | 1696 | 18% | 1721 | 19% | 221 | 13% | 247 | 14% |
| **Socio-economic status (SES) index category based on zip code, n(%)** |  | | | | | | | | | | | | | | | |
| 1 | 4708 | 41% | 4649 | 40% | 1453 | 42% | 1447 | 42% | 3841 | 42% | 3803 | 41% | 719 | 41% | 745 | 42% |
| 2 | 3130 | 27% | 3225 | 28% | 928 | 27% | 921 | 27% | 2499 | 27% | 2537 | 27% | 481 | 27% | 487 | 28% |
| 3 | 2261 | 20% | 2227 | 19% | 622 | 18% | 628 | 18% | 1700 | 18% | 1686 | 18% | 328 | 19% | 296 | 17% |
| 4 | 873 | 8% | 831 | 7% | 239 | 7% | 234 | 7% | 640 | 7% | 653 | 7% | 108 | 6% | 107 | 6% |
| **Prenatal Care, n (%)** |  | | | | | | | | | | | | | | | |
| Prenatal care in first trimester | 6168 | 53% | 6289 | 54% | 2052 | 60% | 2085 | 61% | 4910 | 53% | 4908 | 53% | 929 | 53% | 946 | 54% |

| **Characteristics** | **No Hypertension** | | **Gestational hypertension** | | **No Hypertension** | | **Chronic hypertension** | | **No Hypertension** | | **Preeclampsia** | | **No Hypertension** | | **Superimposed Preeclampsia** | |
| --- | --- | --- | --- | --- | --- | --- | --- | --- | --- | --- | --- | --- | --- | --- | --- | --- |
|  | n / mean | % / SD | n / mean | % / SD | n / mean | % / SD | n / mean | % / SD | n / mean | % / SD | n / mean | % / SD | n / mean | % / SD | n / mean | % / SD |
| **Deyo-Charlson comorbidity index score (mean, SD)** | 0.24 | 0.66 | 0.25 | 0.67 | 0.45 | 1.01 | 0.45 | 0.98 | 0.24 | 0.68 | 0.26 | 0.68 | 0.42 | 0.93 | 0.42 | 0.85 |
| 0, n (%) | 9484 | 82% | 9,354 | 81% | 2409 | 70% | 2,405 | 70% | 7604 | 82% | 7,455 | 81% | 1265 | 72% | 1255 | 72% |
| 1, n (%) | 1763 | 15% | 1,852 | 16% | 753 | 22% | 744 | 22% | 1356 | 15% | 1,444 | 16% | 349 | 20% | 352 | 20% |
| 2, n (%) | 226 | 2% | 256 | 2% | 170 | 5% | 176 | 5% | 185 | 2% | 231 | 3% | 102 | 6% | 99 | 6% |
| 3+, n (%) | 110 | 1% | 121 | 1% | 90 | 3% | 97 | 3% | 89 | 1% | 104 | 1% | 39 | 2% | 49 | 3% |
| **Complications during index pregnancy, n (%)** |  | | | | | | | | | | | | | | | |
| Gestational diabetes | 1877 | 16% | 1,979 | 17% | 679 | 20% | 683 | 20% | 1554 | 17% | 1,623 | 18% | 457 | 26% | 460 | 26% |
| Urinary tract infection | 1758 | 15% | 1,861 | 16% | 676 | 20% | 704 | 21% | 1455 | 16% | 1,550 | 17% | 288 | 16% | 294 | 17% |
| Obesity/overweight | 4321 | 37% | 4,225 | 36% | 1449 | 42% | 1,370 | 40% | 3303 | 36% | 3,180 | 34% | 981 | 56% | 930 | 53% |
| Substance use disorders | 1200 | 10% | 1,292 | 11% | 478 | 14% | 483 | 14% | 920 | 10% | 952 | 10% | 248 | 14% | 235 | 13% |
| Placental previa | 351 | 3% | 411 | 4% | 130 | 4% | 139 | 4% | 286 | 3% | 331 | 4% | 74 | 4% | 78 | 4% |
| **History of hypertension prior to index pregnancy, n (%)** |  | | | | | | | | | | | | | | | |
| More severe hypertension | 479 | 4.1% | 496 | 4.3% | 189 | 6% | 188 | 5% | 518 | 6% | 566 | 6% | 177 | 10% | 181 | 10% |
| Less severe hypertension | 2013 | 17.4% | 2,113 | 18.2% | 1023 | 30% | 1,065 | 31% | 1380 | 15% | 1,435 | 16% | 640 | 36% | 677 | 39% |

| **Characteristics** | **No Hypertension** | | **Gestational hypertension** | | **No Hypertension** | | **Chronic hypertension** | | **No Hypertension** | | **Preeclampsia** | | **No Hypertension** | | **Superimposed Preeclampsia** | |
| --- | --- | --- | --- | --- | --- | --- | --- | --- | --- | --- | --- | --- | --- | --- | --- | --- |
|  | n / mean | % / SD | n / mean | % / SD | n / mean | % / SD | n / mean | % / SD | n / mean | % / SD | n / mean | % / SD | n / mean | % / SD | n / mean | % / SD |
| **Other medical conditions, n (%)** |  | | | | | | | | | | | | | | | |
| History of Type 1 or 2 diabetes | 666 | 6% | 742 | 6% | 453 | 13% | 465 | 14% | 651 | 7% | 692 | 7% | 224 | 13% | 243 | 14% |
| History of renal disease | 66 | 1% | 65 | 1% | 41 | 1% | 45 | 1% | 38 | 0.4% | 54 | 1% | 31 | 2% | 38 | 2% |
| Stroke | 41 | 0.4% | 48 | 0.4% | 31 | 1% | 28 | 1% | 30 | 0.3% | 44 | 0.5% | 15 | 1% | 17 | 1% |
| Pulmonary edema | 31 | 0.3% | 26 | 0.2% | 23 | 1% | 22 | 1% | 32 | 0.3% | 32 | 0.3% | 9 | 1% | 12 | 1% |
| Renal insufficiency | 74 | 1% | 89 | 1% | 44 | 1% | 50 | 1% | 66 | 1% | 82 | 1% | 36 | 2% | 41 | 2% |
| Myocardial infarction | 19 | 0.2% | 24 | 0.2% | 17 | 0.5% | 13 | 0.4% | 17 | 0.2% | 17 | 0.2% | 8 | 0.5% | 13 | 1% |
| History of postpartum hemorrhage | 140 | 1% | 141 | 1% | 51 | 1% | 55 | 2% | 74 | 1% | 90 | 1% | 27 | 2% | 36 | 2% |
| Impaired liver function | 282 | 2% | 340 | 3% | 179 | 5% | 177 | 5% | 242 | 3% | 285 | 3% | 71 | 4% | 89 | 5% |
| Note: Characteristics that do not add up to 100% (ex. region or race) are due to missing or other category. | | | | | | | | | | | | | | | | |

**Supplemental Table 3.** Descriptive statistics for maternal and neonatal clinical outcomes, utilization and cost by maternal hypertensive disorder during pregnancy among Medicaid mothers (compared to mothers with no hypertension).

|  | **No Hypertension** | | **Gestational Hypertension** | | **No Hypertension** | | **Chronic Hypertension** | | **No Hypertension** | | **Preeclampsia** | | **No Hypertension** | | **Superimposed Preeclampsia** | |
| --- | --- | --- | --- | --- | --- | --- | --- | --- | --- | --- | --- | --- | --- | --- | --- | --- |
|  | **n** | **%** | **n** | **%** | **n** | **%** | **n** | **%** | **n** | **%** | **n** | **%** | **n** | **%** | **n** | **%** |
| **All Matched Mothers** | | | | | | | | | | | | | | | | |
| Cesarean delivery | 3741 | 32.3% | 4127 | 35.6% | 1,225 | 35.8% | 1,327 | 38.8% | 3,000 | 32.5% | 4,032 | 43.7% | 693 | 39.5% | 989 | 56.4% |
| Stillbirth | 87 | 0.8% | 110 | 0.9% | 41 | 1.2% | 70 | 2.0% | 83 | 0.9% | 126 | 1.4% | 22 | 1.3% | 26 | 1.5% |
| **Mothers with 12 months continuous enrollment, measured post-delivery** | | | | | | | | | | | | | | | | |
| Postpartum preeclampsia | 23 | 0.5% | 81 | 1.8% | 5 | 0.3% | 27 | 1.7% |  |  |  |  |  |  |  |  |
| Post-delivery  hypertension | 120 | 2.7% | 823 | 18.2% |  |  |  |  | 91 | 2.5% | 635 | 17.3% |  |  |  |  |
| **Liveborn and singleton neonates linked to mothers, measured within 28 days of birth** | | | | | | | | | | | | | | | | |
| Preterm delivery | 285 | 3.0% | 298 | 3.1% | 72 | 2.5% | 124 | 4.5% | 213 | 2.8% | 312 | 4.2% | 36 | 2.5% | 99 | 7.0% |
| Sepsis | 896 | 9.4% | 984 | 10.4% | 269 | 9.5% | 348 | 12.5% | 656 | 8.7% | 1378 | 18.6% | 163 | 11.3% | 419 | 29.7% |
| Respiratory distress syndrome | 779 | 8.2% | 777 | 8.2% | 259 | 9.2% | 348 | 12.5% | 600 | 8.0% | 1777 | 24.0% | 132 | 9.2% | 635 | 45.0% |
| Low birth weight | 458 | 4.8% | 529 | 5.6% | 166 | 5.9% | 198 | 7.1% | 362 | 4.8% | 542 | 7.3% | 78 | 5.4% | 185 | 13.1% |
| Severe congenital defects* | 285 | 3.0% | 298 | 3.1% | 72 | 2.5% | 124 | 4.5% | 213 | 2.8% | 312 | 4.2% | 36 | 2.5% | 99 | 7.0% |

*Severe congenital defects include anophthalmia/microphthalmia, anotia/microtia, craniosynostosis, diaphragmatic hernia, Down Syndrome, esophageal atresia, gastroschisis, hypospadias, microcephaly and upper and lower limb reduction defects.

|  | **No Hypertension** | | | **Gestational Hypertension** | | | **No Hypertension** | | **Chronic Hypertension** | | | **No Hypertension** | | | | **Preeclampsia** | | | | | | **No Hypertension** | | | | | | | **Superimposed Preeclampsia** | | | | |
| --- | --- | --- | --- | --- | --- | --- | --- | --- | --- | --- | --- | --- | --- | --- | --- | --- | --- | --- | --- | --- | --- | --- | --- | --- | --- | --- | --- | --- | --- | --- | --- | --- | --- |
|  | n/  mean | %/SD | | n/  mean | | %/SD | n/  mean | %/SD | n/  mean | | %/SD | | n/  mean | | %/SD | | n/  mean | | | %/SD | | | n/  mean | | | %/SD | | | n/  mean | | | %/SD | |
| **Maternal Post-Discharge Healthcare Utilization and Costs** | | | | | | | | | | | | | | | | | | | | | | | | | | | | | | | | | |
| **Healthcare Utilization** |  | | | | | | | | | | | | | | | | | | | | | | | | | | | | | | | | |
| Hospitalizations | 455 | 10% | | 486 | | 11% | 173 | 12% | 212 | | 14% | | 346 | | 10% | | | 489 | | | 13% | | | 86 | | | 12% | | | 87 | | 12% | |
| Hospitalizations, Count | 0.13 | 0.42 | | 0.15 | | 0.54 | 0.15 | 0.49 | 0.19 | | 0.61 | | 0.12 | | 0.40 | | | 0.19 | | | 0.63 | | | 0.16 | | | 0.50 | | | 0.18 | | 0.79 | |
| ED visits | 2166 | 48% | | 2265 | | 50% | 725 | 48% | 850 | | 55% | | 1688 | | 47% | | | 1864 | | | 51% | | | 357 | | | 49% | | | 402 | | 54% | |
| ED visits, Count | 1.10 | 1.84 | | 1.23 | | 2.11 | 1.11 | 1.84 | 1.51 | | 2.97 | | 1.08 | | 1.98 | | | 1.17 | | | 1.80 | | | 1.25 | | | 2.10 | | | 1.25 | | 1.82 | |
| **Healthcare Cost** |  | | | | | | | | | | | | | | | | | | | | | | | | | | | | | | | | |
| Total Allowed Pharmacy Cost | $746 | $5,075 | | $863 | | $3,553 | $1,293 | $8,832 | $1,306 | $6,206 | | $662 | | $3,507 | | $866 | | | $3,634 | | | $983 | | | | $4,672 | | | $1,332 | | | $5,235 | |
| Total Allowed Medical Cost | $2,788 | $5,586 | | $3,178 | | $6,371 | $3,023 | $4,735 | $4,124 | $8,746 | | $3,033 | | $11,483 | | $3,275 | | | $6,158 | | | $3,349 | | | | $7,672 | | | $3,854 | | | $11,379 | |
| **Neonatal Healthcare Utilization and Costs** | | | | | | | | | | | | | | | | | | | | | | | | | | | | | | | | | |
| NICU admissions |  |  |  | |  | |  |  |  | |  | |  | |  | | |  | | |  | | | |  | | |  | | |  | |  |
| NICU Allowed Cost | $3,114 | $25,749 | $2,927 | | $20,912 | | $4,735 | $42,662 | $4,621 | | $24,911 | | $3,193 | | $26,536 | | | $7,405 | | | $31,133 | | | | $4,657 | | | $35,612 | | | $16,699 | | $60,365 |
| NICU Length of stay, among those with a NICU admission | 14 | 21 | 11 | | 18 | | 15 | 26 | 15 | | 22 | | 13 | | 22 | | | 16 | | | 22 | | | | 14 | | | 23 | | | 22 | | 29 |
